# Risk Factors of HCV Seroconversion in Hemodialysis Patients in Tabriz, Iran

**DOI:** 10.5812/hepatmon.17417

**Published:** 2014-06-01

**Authors:** Mohammad Hossein Somi, Jalal Etemadi, Morteza Ghojazadeh, Sara Farhang, Mehrasa Faramarzi, Sanaz Foroutan, Maryam Soleimanpour

**Affiliations:** 1 Liver and Gastrointestinal Disease Research Center, Tabriz University of Medical Sciences, Tabriz, IR Iran; 2 Chronic Kidney Diseases Research Center, Tabriz University of Medical Sciences, Tabriz, IR Iran; 3 Cliniclal Psychiatry Research Center, Tabriz University of Medical Sciences, Tabriz, IR Iran

**Keywords:** Hepatitis C, Hemodialysis units, Risk Factors, Seroepidemiologic Studies

## Abstract

**Background:**

Hepatitis C virus (HCV) infection is a significant health concern in patients with end-stage renal disease under dialysis. Epidemiological studies have reported a prevalence rate of 5.5-55.9% for this condition in Iran.

**Objectives:**

We evaluated the risk factors for HCV infection and seroconversion in hemodialysis patients.

**Patients and Methods:**

A retrospective analysis was performed on 455 hemodialysis patients from each of the five dialysis units in Tabriz, northwest Iran. Possible risk factors for HCV infection and seroconversion were evaluated.

**Results:**

A total of 37 patients were HCV positive (8.1% of the study population) and seroconversion occurred in 18 of them during the dialysis treatment (3.95% of the study population). History of renal transplantation (44.4%, P < 0.0001), surgical intervention (except for renal transplantation and AV fistula placement) (94.4%, P = 0.03), and mean duration of dialysis (106.06 ± 55.519, P < 0.0001) had strong statistically significant associations with the seroconversion.

**Conclusions:**

The current study indicates increased risk for HCV infection in patients under dialysis and its relation with the mean duration of hemodialysis, history of renal transplantation and surgical intervention. Considering the immune deficiency in these patients, intense education to both patients and medical staff will be beneficial.

## 1. Background

Hepatitis C virus (HCV) infecting 170 million persons worldwide, is a major public health concern (1). Only 20–30% of patients infected with HCV recover spontaneously and the remaining 70–80% develops chronic HCV infection. Regrettably, 3–11% of those patients will develop liver cirrhosis within 20 years, which is a strong risk factor for liver failure and hepatocellular carcinoma (HCC) (2).Injections, transfusion of blood products, organ transplantation, chronic hemodialysis (HD), occupational exposure among health care workers, unprotected sexual contact and vertical transmission are the main risk factors for HCV transmission (3, 4). HCV infection is a serious problem in patients receiving dialysis treatment (5, 6), as they have higher risk of HCV infection causing liver diseases contributing to additional morbidity and mortality in patients with end-stage renal disease (ESRD) (7). Epidemiological studies among hemodialysis patients in Iran have reported a prevalence rate of 5.5-55.9% for HCV in different centers while the reported prevalence rate in the general population in Iran is less than 1% (8-11). The prevalence of ESRD is 242 in one million worldwide and it increases about 8% annually. During the past 30 years, the number of patients with ESRD treated by hemodialysis in the United States has sharply increased (12). Sixty to seventy percent of persons newly infected with HCV are typically asymptomatic or have a mild clinical illness. The majority of infected persons might not be aware of their infection because they are not clinically ill (12). HCV RNA is detectable in blood within 1–3 weeks after exposure (13). The average incidence of anti-HCV seroconversion after accidental percutaneous exposure to an HCV positive source is 1.8% (range 0 to 7%) (14). Eight to nine weeks is the average time from exposure to seroconversion (detectable HCV antibody) and anti-HCV can be detected in > 97% of persons six months later. Infected persons are a source of transmission to others while they are at risk for chronic liver diseases decades after they are infected themselves. These facts are reflected in study by Alter et al. confirming that HD is one of the important risk factors for acquiring HCV infection and that the risk of infection is correlated to the duration of dialysis (15). Furthermore, Fabrizi et al. Hardy et al. and Sandhu et al. found clear evidence for a relation between the time on HD and the number of given transfusions and HCV infection (16-18). Studies continue to find the relation between HCV infection and the related risk factors in different parts of the world to find better ways to prevent additional harm. We previously reported HCV prevalence and the related risk factors in five dialysis units in Tabriz six years ago and noticed that the risk for HCV infection of CRF patients under dialysis would increase with the longer duration and higher frequency of hemodialysis which may be reduced by early transplantation (10). Considering probable epidemiological changes of HCV prevalence in these centers, we followed the previous study in the same centers while collecting additional data.

## 2. Objectives

The aim of the present study was to evaluate the sero-prevalence and risk factors for HCV infection and HCV sero-conversion in all of the patients under dialysis treatment in Tabriz, northwest of Iran.

## 3. Patients and Methods

This retrospective study was carried out in five dialysis units in Tabriz, located in northwest of Iran. The study was approved by the Ethics Committee of Tabriz University of Medical Sciences and informed written consents were signed by all patients.

### 3.1. Study Population

Between December and January 2012, all 455 patients on hemodialysis were invited to take part in this survey. Socio-demographic data were collected by interviewers trained by academic members of the Gastroenterology and Nephrology departments. These data included age, sex, duration of hemodialysis treatment, frequency of dialysis, history of diabetes, blood transfusions, renal transplantation, war injury, surgical interventions (except for renal transplantation and AV fistula placement), multiple sexual partners and possible household acquirement of hepatitis infection. We collected past medical history of patients via patient health records as well, including results of HCV-Ab and HBs-Ag in particular.

### 3.2. Definition of HCV Status

All patients had both a baseline HCV serology and a follow-up HCV serology. The baseline data were gathered from their medical records back to the beginning of dialysis treatment in each patient. Follow up data were also gathered from their records containing the spontaneous check of HCV status. HCV-Ab was checked if no follow up results were available at the time of the study. The diagnosis of hepatitis C infection was verified on the basis of the presence of anti-HCV antibody in the sera detected by third generation enzyme-linked immnosurbent assay (ELISA) kits (Abbott Laboratories, Chicago, USA). Sero-conversion was deﬁned as a change from HCV antibody negative status at the time of initial testing to HCV antibody positive status in the latest report (e.g. a new case of HCV infection).

### 3.3. Statistical Analysis

First, we compared the two groups of HCV positive and HCV negative patients. Then analysis was focused on patients with seroconversion compared to patients with no change in their status, to identify the factors contributing to seroconversion during the dialysis treatment. Descriptive and analytical statistical methods were used throughout data analysis using SPSS version 17. Data are presented as mean± standard deviation or number (percentages). Chi-square test and Fisher’s exact test were used, when appropriate. P ≤ 0.05 was considered as statistically significant. Odds ratios (OR) were calculated considering the confidence interval of 95%.

## 4. Results

**Figure 1.**
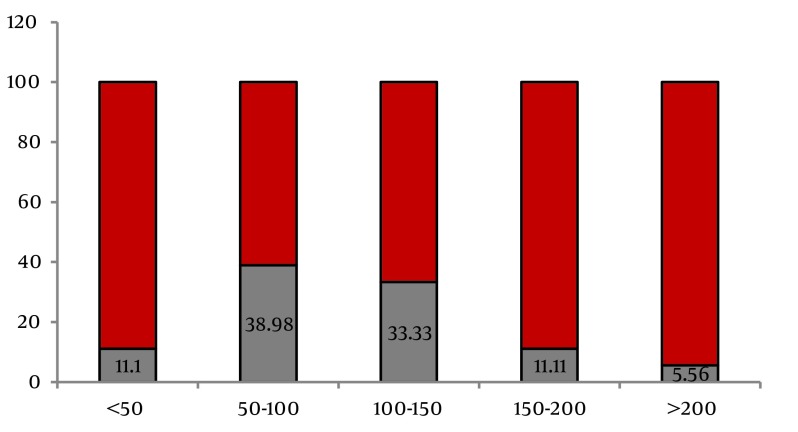
Rate of Seroconversion to Hepatitis C virus Positive Status During Dialysis Divided by Months of Treatment

The age range of the studied population was six to 90 years (mean = 55.98 ± 15.6 years). From the total of 455, 275 (60.4%) were males. Mean duration of dialysis treatment was 43.32 ± 48.84 months (ranging from three to 360 months). Results indicated that 8.1% of the study population were HCV positive. HBS Ag was reported to be positive in 14 (3.07%) patients during the treatment. None of patients was co-infected by HCV and HBV. Table 1 shows the characteristics of the studied population accordingly. HCV infection was significantly higher in patients with younger age [OR = 1.02 (1.00-1.04)], history of blood transfusion [OR = 1.31 (1.12-1.53)], history of renal transplantation [OR = 5.21 (3.33-8.14)] and longer duration of dialysis [OR = 1.07 (0.96- 1.07)] as shown in Table 1. According to another classification, seroconversion occurred in 18 patients out of 455 patients (3.95%) while no changes were observed in HCV infection status of 437 patients. In other words, from the total of 37 HCV-Ab positive patients, 19 (51.3%) were already seropositive at the beginning of the study, while seroconversion occurred in 18 (48.6%) patients during dialysis treatment. Table 1 summarizes the demographic and comorbid characteristics of these two groups as well. Seroconversion was not significantly associated with age, gender or marital status of patients. History of peritoneal dialysis, diabetes, positive HBV contact, number of dialysis sessions per week, multiple sexual partners, and intravenous drug abuse or tattooing were not associated with seroconversion. Seroconversion was higher in patient with a history of blood transfusion but the difference was d not statistically significant. As described in Table 1, a significant association was observed between the risk of seroconversion with a history of renal transplantation [OR = 1.59 (1.05-2.41)] and a history of surgical interventions [(OR = 4.94 (0.73-33.4)]. Mean duration of dialysis was also significantly longer in patients with seroconversion [OR = 1.01 (1.00-1.02)], with medians of 102 months in patients with seroconversion and 25 months in patients with no change in their status. Seroconversion was most likely to occur between 50 and 100 months of dialysis (38.89%) as shown in Figure 1.

**Table 1.** Characteristics of Patients Under Dialysis in Tabriz According to Hepatitis C Virus Infection and Patients With Seroconversion During the Dialysis Treatment

-	HCV ^a^ Status			Seroconversion Status		
	HCV Positive	HCV Negative	P Value	Seroconversion to HCV Positive	No Change	P Value
**Total, No. %**	37 (8.1)	418 (91.9)	-	18 (3.9)	437 (96.1)	-
**Mean Age ± SD**	50.35 ± 12.345	56.48 ± 15.829	0.020	51.11 ± 13.03	56.19 ± 15.73	0.176
**Married**	32 (86.5)	385 (92.3)	0.210	16 (88.9)	401 (91.8)	0.665
**Peritoneal dialysis**	0	29 (6.9)	0.090	0	29 (6.6)	0.254
**Diabetes**	4 (10.8)	167 (63.9)	0.002	3 (16.7)	168 (38.4)	0.163
**Blood transfusion**	31 (83.8)	267 (63.9)	< 0.001	13 (72.2)	285 (65.2)	0.495
**Renal transplantation**	18 (48.6)	39 (9.3)	0.005	8 (44.4)	49 (11.2)	< 0.001
**Contact with HBV** ^**a**^ ** infection**	0	19 (4.5)	0.320	0	19 (4.3)	0.394
**Sexual multi partner**	0	1 (2.7)	0.910	0	1 (2)	0.956
**IVDU** ^**a**^	1 (2.7)	3 (7)	0.213	1 (5.6)	11 (2.5)	0.713
**Tattooing**	1 (2.7)	10 (2.4)	0.900	0	11 (2.5)	0.495
**Surgical intervention**	31 (83.8)	303 (72.5)	0.135	17 (94.4)	317 (72.5)	0.032
**Duration of dialysis**	138.41 ± 80.579	34.90 ± 34.105	< 0.001	106.06 ± 55.519	40.73 ± 46.845	< 0.0001
**Number of dialysis session per week**	-	-	-	-	-	-
One	0	12 (2.9)	0.290	0	15 (3.4)	0.541
More than one	37 (100)	403 (97.1)	0.290	18 (100)	422 (96.6)	0.423

^a^ Abbreviations: HCV, Hepatitis C virus; HBV, Hepatitis B virus; IVDU, Intravenous Drug User.

## 5. Discussion

This retrospective study estimated HCV prevalence and seroconversion rates in hemodialysis patients in five dialysis units and evaluated associations between risk factors and facility-specific practice patterns and HCV prevalence and seroconversion. This is the first study to report on the seroconversionrate of HCV in hemodialysis patients in Tabriz. The seroconversionrate for HCV infection among dialysis patients is generally much higher than healthy people (18, 19) and needs especial attention. This study reports that 8.1% of patients undergoing dialysis were HCV positive and the main risk factors were blood transfusion, renal transplantation and duration of dialysis treatment, while they were younger and the rate of diabetes mellitus was lower among these patients. Seroconversion occurred in 48.9% of HCV positive patients (3.9% of total) and more likely between months 50 to 100 of dialysis treatment. Many patients with ESRD require blood transfusion for treatment of anemia (20, 21) while it is a known risk factor for HCV transmission (22). Medical interventions play the most important role in HCV transmission through blood transfusion or contaminated syringes and needles (23). Compatible with the current results, these factors were also a significant risk factor in our previous study performed in 2006 in Tabriz (11). These results vary between different centers and the relation between HCV seropositivity and history of blood transfusion in dialysis patients is reported in some (24, 25) but not all of these reports (17, 26). Positive history of blood transfusion was higher in patients with seroconversion but the difference was not statistically significant.

The same results were obtained for history of war-related injury and a positive result of HCV antibody and seroconversion. Similar results are reported by Sead Ahmetagic et al. who did not find any association between the war-related injury and HCV infection (11, 27). The duration of dialysis treatment could be a specific, independent risk factor for HCV infection. Similar to our results in 2006, that the prevalence of HCV in hemodialysis patients was associated with the longer duration of the treatment, we again found that HCV positive patients had a longer duration of treatment and the duration of dialysis was also related to seroconversion. These results are compatible with the results of other studies (17, 24, 25,28-30). Although HCV infection is an independent risk factor for increased mortality and also for graft loss after renal transplantation, renal transplantation remains the best therapeutic option for HCV positive patients with ESRD (30). In the current study, there were significant associations between seroconversion with history of renal transplantation and surgical intervention (except for renal transplantation and AV fistula placement). But we just found statistically significant relation between HCV positive status and renal transplantation but not other surgical interventions. These findings are compatible with the results of other studies (11). This may indicate the extremely high rate of transmitted infections like HCV in the immune suppressed transplant recipients. In addition, high percentage of surgical intervention (94.4%) in seroconversion group in comparison with (72.5%) the negative group can be investigated in further studies in the future. Some previous reports showed the higher incidence and prevalence of type 2 diabetes mellitus, one of the most common causes of chronic renal failure, in HCV-infected patients in comparison with the general population (31, 32). Diabetes mellitus, chronic renal failure, and HCV infection all can increase the morbidity and mortality in patients, also can impair quality of life (33). In our study, diabetes mellitus was significantly lower in HCV positive compared to HCV negative patients as well as patients with seroconversion. This result could be due to the lower mean age of the HCV positive patients or may be explained by higher education provided to these patients. The main limitation of this study is that seroprevalence of HCV was not confirmed by the detection of HCV RNA in blood samples We also report that seroconversion was most likely to occur between months 50 to 100 of dialysis treatment. However serologic evaluations were not done regularly in all of patients, so these results is biased as the infection could be detected later. .In conclusion, the rate of HCV positive patients with ESRD in dialysis centers in our region has decreased from the rate observed in 2006 (14.9%) (11), but still is related to the same risk factors and needs special consideration. As a majority of patients newly infected with HCV are typically asymptomatic, careful and regular assessments aimed at patients with bearing the above risk factors can be beneficial. Because of the important role of blood transfusion, behaviors like changing gloves for each patient, paying careful attention to hygiene, complete sterilization of dialysis machines, and decreasing the use of blood products to the least possible extent are recommended to prevent transmission of all blood-borne pathogens including HCV (34). The strong association between duration of hemodialysis and seroconversion could be because of an increased possibility of exposure to the above mentioned factors. Providing hemodialysis patients with more facilities including earlier transplantations will be beneficial in this regard.
